# Characterization of the L-Arginine/Nitric Oxide Pathway and Oxidative Stress in Pediatric Patients with Atopic Diseases

**DOI:** 10.3390/ijms23042136

**Published:** 2022-02-15

**Authors:** Beatrice Hanusch, Kathrin Sinningen, Folke Brinkmann, Stefanie Dillenhöfer, Mirjam Frank, Karl-Heinz Jöckel, Cordula Koerner-Rettberg, Martin Holtmann, Tanja Legenbauer, Christian Langrock, Thomas Reinehr, Patricia Maasjosthusmann, Bibiana Beckmann, Eckard Hamelmann, Dimitrios Tsikas, Thomas Lücke

**Affiliations:** 1 University Hospital of Pediatrics and Adolescent Medicine, St. Josef-Hospital, Ruhr-University Bochum, 44791 Bochum, Germany; beatrice.hanusch@rub.de (B.H.); kathrin.sinningen@rub.de (K.S.); folke.brinkmann@rub.de (F.B.); stefanie.dillenhoefer@klinikum-bochum.de (S.D.); 2 Institute for Medical Informatics, Biometry and Epidemiology, University of Duisburg-Essen, 45122 Essen, Germany; mirjam.frank@uk-essen.de (M.F.); k-h.joeckel@uk-essen.de (K.-H.J.); 3 Clinic for Children and Youth Medicine, Marien-Hospital, 46483 Wesel, Germany; cordula.koerner-rettberg@prohomine.de; 4 LWL University Hospital for Child and Adolescent Psychiatry, Ruhr University Bochum, 44791 Bochum, Germany; martin.holtmann@lwl.org (M.H.); tanja.legenbauer@rub.de (T.L.); 5 Department of Pediatric Endocrinology, Diabetes and Nutrition Medicine, Vestische Kinder- und Jugendklinik Datteln, University of Witten/Herdecke, 45711 Datteln, Germany; info@psychotherapie-langrock.de (C.L.); t.reinehr@kinderklinik-datteln.de (T.R.); 6 University Children’s Center Bethel, Protestant Hospital Bethel, University Bielefeld, 33617 Bielefeld, Germany; patricia.maasjosthusmann@evkb.de (P.M.); eckard.hamelmann@evkb.de (E.H.); 7 Institute of Toxicology, Core Unit Proteomics, Hannover Medical School, 30623 Hannover, Germany; beckmann.bibiana@mh-hannover.de (B.B.); tsikas.dimitros@mh-hannover.de (D.T.)

**Keywords:** atopic diseases, nitric oxide, attention deficit/hyperactivity syndrome, bronchial asthma, atopic dermatitis, common pediatric diseases

## Abstract

Introduction: L-Arginine (Arg) is a semi-essential amino acid. Constitutive and inducible nitric oxide synthase (NOS) isoforms convert Arg to nitric oxide (NO), a potent vaso- and bronchodilator with multiple biological functions. Atopic dermatitis (AD) and bronchial asthma (BA) are atopic diseases affecting many children globally. Several studies analyzed NO in airways, yet the systemic synthesis of NO in AD and BA in children with BA, AD or both is elusive. Methods: In a multicenter study, blood and urine were obtained from 130 of 302 participating children for the measurement of metabolites of the Arg/NO pathway (BA 31.5%; AD 5.4%; AD + BA 36.1%; attention deficit hyperactivity disorder (ADHD) 12.3%). In plasma and urine amino acids Arg and homoarginine (hArg), both substrates of NOS, asymmetric dimethylarginine (ADMA) and symmetric dimethylarginine (SDMA), both inhibitors of NOS, dimethylamine (DMA), and nitrite and nitrate, were measured by gas chromatography–mass spectrometry. Malondialdehyde (MDA) was measured in plasma and urine samples to evaluate possible effects of oxidative stress. Results: There were no differences in the Arg/NO pathway between the groups of children with different atopic diseases. In comparison to children with ADHD, children with AD, BA or AD and BA had higher plasma nitrite (*p* < 0.001) and nitrate (*p* < 0.001) concentrations, suggesting higher systemic NO synthesis in AD and BA. Urinary excretion of DMA was also higher (*p* = 0.028) in AD and BA compared to patients with ADHD, suggesting elevated ADMA metabolization. Discussion/Conclusion: The Arg/NO pathway is activated in atopic diseases independent of severity. Systemic NO synthesis is increased in children with an atopic disease. Plasma and urinary MDA levels did not differ between the groups, suggesting no effect of oxidative stress on the Arg/NO pathway in atopic diseases.

## 1. Introduction

Atopic diseases such as bronchial asthma (BA) and atopic dermatitis (AD) are among the most prevalent chronic diseases. In Germany, the life-time prevalence of atopic disease in children and adolescents is 23.7%, with a steep increase worldwide over the last two decades [1,2,3,4].

The underlying mechanism of atopic diseases is a chronic inflammation. In allergic asthmatic patients, this inflammation can be assessed with the biomarker of fractional exhaled Nitric Oxide (FENO), which typically is increased in the exhaled air [5]. Nitric Oxide (NO) is important for lung function, as it is not only vasodilative but also involved in bronchodilation [6]. The American Thoracic Society (ATS) recommends the measurement of FENO for eosinophilic airway inflammation diagnosis and to evaluate the likelihood of steroid treatment response in patients with chronic respiratory symptoms. However, ATS only suggests usage of FENO as a supportive measure in BA diagnosis [7]. In children, the implementation of FENO measurement seems specifically promising as it is a non-invasive, easy-to-obtain and sensitive measure, which could help to reduce the amount of exacerbations in patients [8]. In BA patients, the L-Arginine (Arg)/NO pathway seems to be associated to the disease status. Kraj et al. observed lower Arg and higher L-citrulline and L-ornithine concentrations in serum of patients with well-controlled BA than in healthy controls [9], while Morris et al. found lower Arg, L-citrulline and L-ornithine in more exacerbated cases of BA patients between 2 and 52 years old compared to healthy controls [10]. Accordingly, 232 BA patients (12.9% below 18 years old) from a different study showed elevated arginase activity with lower L-ornithine compared to healthy controls. The Arg bioavailability and arginase activity were associated to the amount of airflow limitation in these patients [6,11,12,13]. In contrast to BA, changes in Arg/NO metabolism in AD have not been well documented and understood until now [14,15,16].

**Figure 1:**
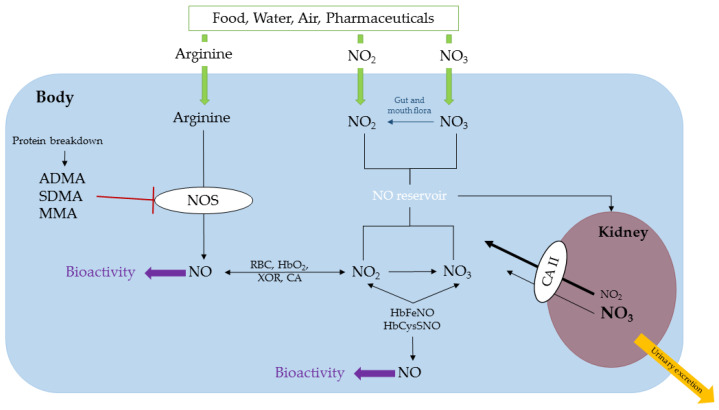
Simplified schematic of the L-arginine/nitric oxide pathway. L-Arginine (Arg) is a semi-essential amino acid. Arg undergoes multiple metabolism including cellular oxidation to nitric oxide (NO) by nitric oxide synthase (NOS) isoforms: endothelial NOS (eNOS), neuronal NOS (nNOS) and inducible NOS (iNOS). Post-translational modification (PTM) of Arg residues in certain proteins yields monomethylarginine (MMA), symmetric dimethylarginine (SDMA) and asymmetric dimethylarginine (ADMA), which inhibit NOS activity. NO is metabolized in red blood cells (RBC) by oxyhemoglobin (HbO_2_) to nitrate, nitrite, nitrosyl hemoglobin (HbFeNO) and S-nitroso-cysteinyl hemoglobin (HbCysSNO). NO, HbFeNO and HbCysSNO are biologically active. Nutrition and some pharmaceuticals can contribute to nitrate and nitrite, which form a cycle that includes reduction of nitrate to nitrite by bacterial nitrate reductase in mouth and gut flora. Circulating nitrate and nitrite are excreted in the urine by several mechanisms including renal carbonic anhydrase II (CA II). XOR, xanthine oxidoreductase, hemoglobin species and CA can convert nitrite to NO. For simplicity, the formulas of nitrate and nitrite are reported without their negative charge.

Arg is a semi-essential amino acid and L-Homoarginine (hArg) is its methylene homologue, produced by the catalytic action of arginine:glycine amidinotransferase (AGAT) [17,18]. In blood, the molar ratio of Arg-to-hArg is about 20:1 to 50:1 [18]. Low circulating hArg concentrations are associated with all-cause mortality and cardiovascular diseases [19]. Constitutive and inducible nitric oxide synthases (NOS) convert Arg and hArg to nitric oxide (NO) [18,20,21] (Figure 1). NO is one of the most potent endogenous vasodilators with multiple additional biological functions such as involvement in the immune system, cancer formation or cancer prevention, and in neurotransmission [18,20,21,22,23]. As NO is a radical gas, which undergoes multiple rapid reactions in biological samples [24], it is not directly measurable. Nitrate and nitrite are major and stable metabolites of NO [25,26,27] (Figure 1). Circulating nitrite and nitrate are useful measures of systemic NO synthesis. The nitrate/nitrite/NO cycle serves as an NO reservoir (Figure 1). Nitrite and nitrate are mainly excreted in the urine [28] (Figure 1). Nitrite and nitrate, to a lesser degree, are reabsorbed from the primary urine mainly by the catalytic action of the renal carbonic anhydrase. Urinary nitrite and nitrate are useful measures of whole-body NO synthesis [29].

Enzyme-catalyzed post-translational modification of the guanidine group (*N*^G^) of Arg residues in proteins and their subsequent hydrolysis produce three methylated Arg analogues: *N*^G^-monomethylarginine (MMA), asymmetric *N*^G^,*N*^G^-dimethylarginine (ADMA), and symmetric *N*^G^,*N*^G^-dimethylarginine (SDMA), which are inhibitors of NOS activity [30,31,32] (Figure 1). ADMA is hydrolyzed by dimethylarginine dimethylaminohydrolase (DDAH) to produce dimethylamine (DMA) and L-citrulline [33,34]. Urinary DMA is the major metabolite of ADMA and a measure of the whole-body asymmetric dimethylation of Arg residues in proteins [35,36].

Imbalances in the Arg/NO pathway have been connected to certain diseases in the cardiovascular, renal, neuronal and immune systems [23,37]. Considerable differences in the Arg/NO pathway are also found between healthy children and adults. For instance, ADMA concentration in plasma and urine were found to decrease from infancy to adulthood [38]. In pediatric patients with inborn metabolic diseases, diabetes type I or attention deficit hyperactivity disorder (ADHD), the Arg/NO pathway was found to differ compared to healthy age-matched children [39,40,41,42,43].

Since NO seems to be involved in the progression of atopic diseases [44], quantitative determination of the status of the Arg/NO pathway in children with AD, BA or BA + AD is of particular interest. We, therefore, included and investigated the Arg/NO pathway in the NIKI Study in pediatric patients with AD, BA and AD + BA. In a sub-cohort of the NIKI Study [45,46,47], we measured several metabolites of the Arg/NO pathway in plasma and urine samples in order to evaluate potential associations of this pathway with atopic diseases. Children with ADHD only served as a control group for patients with atopic diseases, as the NIKI study included ADHD (see below). As obtaining consent for drawing blood from healthy children is quite difficult, no healthy controls were included in the study. Furthermore, patients with atopic diseases have a 30–50% greater chance of developing ADHD later in life; differences between patients affected by atopic diseases without ADHD and such affected by ADHD but not by atopic diseases are of interest [48]. In our study, we assessed oxidative stress by measuring malondialdehyde (MDA) in plasma and urine samples, in order to evaluate possible effects of oxidative stress on NO bioavailability.

## 2. Results

**Figure 2:**
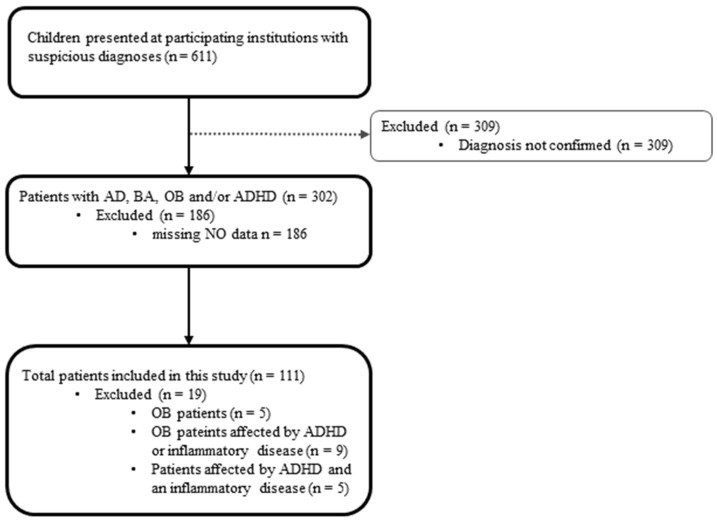
Flowchart of recruitment and inclusion of pediatric patients into this analysis. Abbreviations. BA, bronchial asthma; AD, atopic dermatitis; ADHD, attention deficit/hyperactivity disorder; NO, nitric oxide; OB, obesity.

Overall, the Arg/NO metabolism was analyzed in 130 children participating in the NIKI study. Fourteen participants were excluded due to obesity (Figure 2). Of these, five were affected by obesity only, nine were additionally affected by at least one of the other diseases. Five ADHD patients were excluded from analysis due to being affected by at least one of the inflammatory diseases (BA, AD or the combination of BA + AD). 

The 111 patients (75 boys, 36 girls) included in the analysis were between 6 and 12 [9 (8–10)] years old. Of these patients, 41 were affected by BA, 7 by AD, 47 by BA and AD, and 16 by ADHD (Table 1). Higher IgE serum levels than the age-appropriate threshold were detected in 70.7% of patients affected by BA, 100% affected by AD, 95.7% affected by combined BA and AD and 62.5% of patients affected by ADHD (Table 1).

### 2.1. The Arg/NO Pathway in the Inflammatory Diseases

As atopic diseases tend to progress in life, and underlying mechanisms of BA alone might differ from patients affected by both AD and BA, these groups were further analyzed. As summarized in Table 2, no differences in the Arg/NO pathway were seen between patients only affected by BA and patients affected by both atopic diseases. Patients affected by AD alone were excluded from the main analysis, due to the small sample size. However, additional comparison of AD with BA and BA + AD did not show any significant difference either (Table S1). Also, no differences in the Arg/NO pathway were observed between well-controlled and not-well-controlled BA (Table S2), or between severe and moderate AD (Table S3).

### 2.2. Comparison between ADHD and Type of Inflammatory Disease

Patients affected by ADHD had significantly lower concentrations of nitrate (by 37%) and nitrite (20%) in plasma than patients affected by atopic diseases: BA, AD or BA and AD (nitrate *p* < 0.001; nitrite *p* < 0.001, Table 3). Furthermore, P_NOx_R (by 16%) was significantly lower in ADHD patients than in patients affected by atopic diseases (*p* = 0.001). Patients with atopic diseases had higher excretion rates of nitrate (by 17%) and nitrite (by 18%). Yet, these differences did not reach statistical significance. Patients affected by inflammatory diseases excreted significantly higher DMA concentrations in urine (by 11%) than patients affected by ADHD (*p* = 0.028), while the DMA/ADMA molar ratio only showed a tendency towards higher urinary excretion in patients affected by inflammatory diseases. None of the other Arg/NO parameters showed any difference between the two groups. Patients affected by inflammatory diseases had significantly more often IgE values above the age-appropriate threshold than patients affected by ADHD (*p* = 0.039).

### 2.3. The Arg/NO Pathway and Allergy

As 81 out of the 111 patients of the NIKI study had at least one specific allergic sensitization, defined as at least one specific IgE serum level over 3.5 U/mL, the Arg/NO pathway was analyzed with regard to allergy as well. Nitrate (by 20%; *p* < 0.001) and nitrite (by 12%; *p* < 0.001) were higher in plasma of patients with allergic sensitization compared to those without allergy, while the DMA/ADMA ratio in urine tended to be higher (*p* = 0.053, Table 4). IgE serum levels did not correlate with nitrate or nitrite in plasma and urine (Table S4).

### 2.4. The Arg/NO Pathway and Allergy in Patients Affected by BA, AD or BA and AD

As allergy was defined as the presence of at least one specific IgE over 3.5 U/mL, most patients with allergy were either affected by BA (*n* = 31), AD (*n* = 4) or BA and AD (*n* = 43). Therefore, an additional sub-analysis of the Arg/NO pathway was performed in these groups with regard to allergy. Here, plasma nitrite concentration was found to be significantly higher (by 7%) in patients with allergy compared to patients without allergy (Table 5). The Arg/ADMA ratio was smaller (by 17%) in allergic patients than in those without allergy (*p* = 0.037), plasma Arg concentration tended to be lower in patients with allergy, while none of the other metabolites differed significantly.

## 3. Discussion

Our data show two main findings: Firstly, we observed higher plasma concentrations of nitrite (by 20%) and nitrate (by 37%) in patients with atopic diseases, BA and/or AD, compared to patients with ADHD. Secondly, in the overall test population, patients with increased specific IgE serum levels had significantly higher plasma nitrate concentrations (by 20%) than patients without allergic sensitization. Higher plasma concentrations of nitrite (by 12%) in AD, BA and AD + BA patients with increased specific IgE serum levels were still observed, when ADHD as a co-morbidity was excluded from the analysis. In the sub-analysis of only atopic patients (AD and/or BA), plasma nitrite concentrations were significantly higher (by 7%) in patients with increased specific IgE serum levels compared to atopic patients without signs of specific allergic sensitization. Except for DMA, no significant differences were observed in other metabolites of the Arg/NO pathway or in MDA, a biomarker and measure of oxidative stress [49]. 

Several factors may enhance circulating and excretory nitrite and nitrate in humans (Figure 1). (a) Arginine bioavailability might be elevated, and NOS could therefore be more active. (b) The activity of NOS might be increased due to reduced inhibition by ADMA and therefore heightened nitrate and nitrite concentrations [50,51]. (c) Nitrate and nitrite excretion through kidneys is reduced. Or (d) More nitrate- and nitrite-rich foods or drugs are consumed.

Small changes in Arg homeostasis due to the conversion of Arg to NO are hard to detect even under conditions of high-dosed Arg administration [52]. We did not find higher plasma Arg concentration in atopic patients than in ADHD patients, thus NO production was not increased in these patients due to higher substrate availability, as plasma Arg is rate limiting for whole-body NO synthesis [53]. The major Arg-involving pathways including arginase-catalyzed hydrolysis to L-ornithine are not affected in atopic diseases. With regard to the Arg/NO pathway and considering the relatively low *K*_M_ values (low µM-range) of Arg for all NOS isoforms [54], as well as the relatively high circulating Arg concentrations in the pediatric patients of our study, the Arg bioavailability should be high enough for saturation of all NOS isoforms including iNOS in all NOS-expressing cells. As hemoglobin in venous erythrocytes can react with NO to produce nitrosylated hemoglobin, which could reflect bioavailability of NO in the vascular system and does respond to post-occlusion hyperemia and is correlated with endothelial functioning in humans, it cannot be excluded that our observed changes might be influenced by hemoglobin [55,56]. Regrettably, erythrocyte hemoglobin concentration was not analyzed in participating patients.

Arg and ADMA compete for binding to the NOS; therefore elevated ADMA relative to Arg concentrations in plasma leads to inhibition of the NO producing enzyme [57]. We did not observe a difference in ADMA concentrations between ADHD and atopic patients. Hence, higher nitrate and nitrite concentrations in plasma of atopic patients cannot be explained by a decreased inhibition of NOS activity by ADMA. However, we observed a lower Arg/ADMA ratio in atopic patients with allergic sensitization compared to atopic patients without allergies. Accordingly, NOS activity is not stronger inhibited in patients with atopy compared to patients with ADHD, but allergic sensitization is associated with a stronger inhibition of NOS. This might indicate activation of a negative feedback-loop in allergic atopic patients, as nitrite in plasma was still significantly higher in the atopic patients with allergies than in those without allergies, representing no actual inhibition of NOS. 

Nitrate and nitrite are mainly excreted through urine, while a negligible amount of about 2% of consumed nitrate and nitrite is found in faeces [58]. Reduction of nitrate and nitrite excretion could add into elevation of nitrate and nitrite pool in plasma. As we did not observe differences in urinary content of nitrate and nitrite in the participating children, the higher plasma content of these two molecules cannot be explained by reduced excretion of these NO metabolites.

Nitrate and nitrite are absorbed from foods and drinks. Here, a gastro-oral cycle of nitrate has been described, in which nitrate is absorbed by the stomach, released in the oral cavity via saliva and bacterially converted to nitrite, which is then either absorbed or metabolized in the stomach [59]. McDonagh et al. and Govoni et al. additionally reported increased plasma nitrate and nitrite levels after ingestion of beetroot juice or sodium nitrate, which was attenuated after participants used antibacterial mouthwash [60,61]. The differences we observed in nitrate and nitrite levels in atopic patients and ADHD patients could theoretically be influenced by their oral microflora as well as their diet. As we did observe higher plasma nitrite and nitrate in atopic patients, nitrate-nitrite-NO metabolism could have gained importance in these patients, promoting NO production without the need of elevated Arg concentrations. Unfortunately, usual food intake was not documented in the participants of our study.

As we observed higher nitrite in plasma of allergic atopic patients than in atopic patients without allergic sensitization, involvement of differential NOS activity is possible. As the Arg/ADMA ratio was significantly reduced in the allergic atopic group as well, NOS activity might be inhibited in this group, thus counteracting the heightened activity of NOS during allergic immune reactions. As stated already earlier, ADMA elevation could also be interpreted as part of a negative feedback-loop due to elevated NOS activity. Additionally, as described in depth by DeMartino et al., nitrite can be used to synthetize NO by various metalloproteins [27]. Nitrite itself originates from either NO oxidation or nitrate reduction in salivary glands; thus, nitrate is regarded as a nitrite pool, while nitrite itself functions as a NO pool [27,62]. Therefore, patients with atopic diseases may have a higher NO pool than patients with ADHD. Accordingly, the observed elevated nitrite concentration in allergic atopic patients might serve as a measure of two ways for elevation of a NO supply and a lower Arg/ADMA ratio might serve as an activated negative feedback-loop.

Fernando et al. observed higher nitrate and NOx (sum of nitrate and nitrite in serum) in pediatric patients affected by asthma than in healthy controls [63]. Like ourselves, they observed no differences in nitrate and nitrite depending on the level of asthma control. In contrast to the results in pediatric patients affected by asthma, in patients affected by AD, an association between the higher serum nitrate concentrations and the more severe AD was observed [14].

Additionally, Fernando et al. found a significantly negative correlation between total antioxidant capacity and nitrate and NOx, linking low total antioxidant capacity in pediatric BA patients to higher nitrate and NOx serum concentrations [63]. Omata et al. observed lower nitrate and nitrite concentrations in urine of pediatric AD patients than in healthy controls, while for 8-hydroxy-2′-deoxyguanosine, a marker of oxidative damage of DNA, concentration in urine was higher in these AD patients than in controls [15]. Tsukahara et al. observed elevated oxidative stress in lipids, DNA and bilirubin activity as antioxidant, but no changes in NO production measured in urine during exacerbation in pediatric AD patients and compared to healthy controls [16]. Elevated NOS activity due to acceleration of NO oxidation through abundance of superoxide may lead to the observed higher nitrate and nitrite in plasma under reduced antioxidant capacity. As we did not find differences in MDA, a marker of oxidative stress [49], between the analyzed groups, an influence of oxidative stress on NO oxidation to nitrate and nitrite, seems less likely.

Patients with AD have a higher tendency to also suffer from ADHD, but it is not yet clear whether there is a causal relationship between the two diseases [48]. Therefore, future studies on patients with AD with and without co-morbid ADHD, examining the Arg/NO metabolism, seem intriguing. In addition, it is conceivable that successful treatment of the underlying atopic diseases could contribute to ADHD symptom relief, but further studies are required to show this on a mechanistic basis.

### Limitations

Although a quite large group of patients affected by BA with or without co-morbid AD was included in our study, only a few patients with ADHD or AD ultimately participated in the study. Additionally, as obtaining consent for drawing blood from healthy children is quite difficult, no healthy controls were included in the study. Interestingly, Jansen et al. did observe higher plasma nitrite concentrations in pediatric patients affected by ADHD compared to healthy controls [43]; therefore, inclusion of healthy controls would be desirable in future studies analyzing changes in the Arg/NO metabolism of patients with atopic diseases.

As described by Rao and Phipatanakul [8], FENO measurement in pediatric BA can be used to measure disease progress; it thus would have been quite interesting to analyze differences in whole-body vs. lung-specific Arg/NO metabolism, but sadly FENO was not obtained from the patients included into this study. As diet also influences the nitrate and nitrite concentrations, data on usual foods consumed by patients would have been of interest. 

## 4. Materials and Methods

### 4.1. The NIKI Study

The NIKI study was conducted to analyze atopic diseases, ADHD and overweight in children and adolescents and was performed between 1 October 2013 and 30 September 2016. The multicenter study was executed at the Department of Pediatric Endocrinology, Diabetes and Nutrition Medicine, Vestische Kinder- und Jugendklinik Datteln, University Witten/Herdecke, the University Children’s Hospital, St. Josef Hospital Bochum, Ruhr University Bochum, the LWL University Hospital for Child and Adolescent Psychiatry in Hamm, Ruhr University Bochum, and the LVR-Klinikum Essen, Clinic for Psychiatry, Psychosomatics and Psychotherapy of Childhood and Adolescence, Clinics/Institute University of Duisburg-Essen, Germany. Written informed consent was obtained from all children and their parents. The study was approved by the local ethics committee of the University of Bochum.

The patients recruited were between 6 and 12 years old with overweight, ADHD, and the atopic diseases BA and AD. A total of 611 children (407 boys and 204 girls) were screened and 302 patients (191 boys (63.2%) and 111 girls (36.7%)) were included in the NIKI study. For the 309 excluded subjects, initial diagnosis could not be confirmed (Figure 2). The NIKI study analyzed multiple variables such as anthropometrics, the parent version of the Connors 3^®^ rating scales [64] and blood parameters. The Arg/NO pathway was evaluated in a subset of 130 children. Body height and weight were measured and SDSLMS body mass index (BMI) based on German population-specific data was used to define overweight as BMI >90th age- and gender-dependent percentiles [65,66]. 

For ADHD evaluation, participants were asked to abstain from ADHD medication (e.g., methylphenidate or atomoxetine) at least 48 h prior to testing. Lifetime ADHD was ascertained via previous medical records and diagnoses. Participants with abnormal scores on the Conners 3^®^ scales (“Inattention” or “Hyperactivity/ Impulsivity”) subsequently participated in the ADHD diagnostic module including a semi-structured expert interview (diagnostic checklist, DCL-ADHS) of the Diagnostic System for the Assessment of Mental Disorders in Children and Adolescents based on ICD and DSM (DYSYPS-II) and the parent and teacher rating scales for ADHD (FBBADHS) [67,68,69]. Additionally, a neuropsychological test battery (subtests Go/NoGo and Alertness of the TAP [70]) was conducted. 

Asthma control was evaluated by asthma control test, according to the manufacturer’s guidelines (GlaxoSmithKline Group, Middlesex, UK) [71,72]. Children under the age of 12 filled in a questionnaire by themselves, which was accompanied with a short questionnaire for parents or guardians. Patients 12 years or older were asked to answer the asthma control test for adults. As per manufacturer’s guideline [71,72], all participants were classified as “well-controlled” with 21 points or higher and as “not-well-controlled” with 20 points or lower.

Severity of AD was graded by SCORAD.

### 4.2. Blood and Urine Sampling and Biochemical Analyses

Venous blood samples were collected in ethylenediaminetetraacetic acid (EDTA) monovettes as well as in serum monovettes. The inflammation markers immunoglobulin (Ig) M, IgG, IgE, C-reactive protein (CRP) and interleukin 6 (IL-6) were measured in serum by clinical chemistry laboratory analysis and classified according to age-appropriate cut-points into normal values and increased levels [73]. Allergy was defined as specific IgE over 3.5 U/mL. It was screened for *Aspergillus fumigatus*, dog dander, cat epithelium, rye grass, timothy grass, giant ragweed, alder, *Cladosporium herbarum*, birch, mugwort, *Dermatophatoides microceras*, codfish, cow’s milk, egg white, peanut, soybean, wheat, almond, brazil nut, coconut and hazelnut.

Plasma was generated by centrifugation (4000× *g*, 10 min, 4 °C). Midstream urine samples were collected in the morning. Plasma and urine samples were stored at −80 °C until further analysis. 

Nitrate, nitrite, creatinine and MDA in plasma and urine samples were determined simultaneously by gas chromatography–mass spectrometry (GC–MS) as described previously [74]. Arg and ADMA in plasma and urine were analyzed with GC–MS as described elsewhere [75]. Urinary SDMA and DMA were measured by GC–MS as described [76,77]. MDA served as a measure of oxidative stress [49]. The urinary excretion of all analytes was corrected for creatinine excretion and these data are reported as µM analyte per mM creatinine. 

The nitrate-to-nitrite molar ratios in plasma (P_NOx_R) and urine (U_NOx_R) were determined by dividing the concentration of nitrate in plasma (P_NO3_) or urine (U_NO3_) by the concentration of nitrite in plasma (P_NO2_) or urine (U_NO2_) (i.e., P_NOx_R = P_NO3_/P_NO2_; U_NOx_R = U_NO3_/U_NO2_) [29]. The sum of SDMA, DMA and ADMA in urine was used as measure for whole-body arginine dimethylation, while the ADMA and DMA sum in urine served as a measure of the whole-body asymmetric arginine demethylation [35,36]. The ADMA + DMA/SDMA ratio in urine is a useful measure of the asymmetric to symmetric arginine dimethylation balance in the body [35,36]. 

### 4.3. Statistical Analysis

Statistical analyses were performed using the statistical software package IBM^©^ SPSS^©^ Statistics for Windows, version 25.0 (IBM Corp., Armonk, NY, USA). Descriptive data were analyzed by the Chi-squared test or for groups smaller than five observations by Fisher’s exact test. The Kolmogorov–Smirnov test was used to test for normal distribution. Normally distributed data were analyzed using parametric tests (Student’s *t*-test, one-way ANOVA). Non-normally distributed data were analyzed using non-parametric tests (Mann–Whitney test, Kruskal–Wallis test). Bonferroni post hoc analysis was applied to ANOVA and the Kruskal–Wallis test. Correlations were performed after Pearson for normally distributed data and after Spearman for non-normally distributed data. Values of *p* < 0.05 were considered significant. Data are presented as mean ± standard deviation (SD) or median (25–75th interquartile range). 

## 5. Conclusions

In conclusion, we observed higher plasma nitrate and nitrite levels in pediatric patients affected by one or two atopic diseases than in pediatric patients affected by ADHD. These findings were most likely the result of an elevated NOS activity due to higher inflammatory activity in atopic pediatric patients compared to patients with ADHD.

## Figures and Tables

**Table 1:** Anthropometric characteristics of the patients separated by diagnosis and inflammatory markers.

Parameter/Disease		BA	AD	BA + AD	ADHD	*p* Value
Number of patients		41	7	47	16	
Sex	Male (%)	33 (80.5)	5 (71.4)	27 (57.4)	10 (62.5)	0.120 ^a^
	Female (%)	8 (19.5)	2 (28.6)	20 (42.6)	5 (37.5)	
Age		9 (8–11)	7 (7–10)	9 (7–11)	9 (8–9)	
BMIp		54.5 ± 24.4	not determined	53.4 ± 30.0	72.5 ± 28.8	
Inflammatory markers						
Elevated CRP (%)		2 (5.1)	0 (0)	3 (6.4)	0 (0)	0.890 ^a^
Elevated IL-6 (%)		1 (2.4)	0 (0)	3 (6.4)	0 (0)	0.734 ^a^
Elevated IgM (%)		0 (0)	0 (0)	3 (6.4)	1 (6.3)	0.255 ^a^
**Elevated IgG (%)**		**1 (2.4)**	**2 (28.6)**	**1 (2.1)**	**3 (18.8)**	**0.037 ^a^**
**Elevated IgE (%)**		**29 (70.7)**	**7 (100)**	**45 (95.7)**	**10 (62.5)**	**0.001 ^a^**

Abbreviations. BA, bronchial asthma; AD, atopic dermatitis; ADHD, attention deficit/hyperactivity disorder; BMIp, body mass index percentile; IL-6, interleukin 6; Ig, immunoglobulin; CRP, c-reactive protein; ^a^ Fisher’s exact test. Data are presented as mean ± standard deviation (SD, normally distributed data) or median (25–75th interquartile range, non-normally distributed data). Bold *p* values indicate statistical significance.

**Table 2:** The Arg/NO pathway status in the pediatric patients affected by BA or by BA and AD.

	BA	BA and AD	*p* Value
Number of subjects	41	47	-
Plasma			
Arg (µM)	84.1 (71.2–99.1)	81.7 (67.0–91.5)	0.382
hArg (µM)	1.14 ± 0.49	1.11 ± 0.29	0.735
ADMA (µM)	0.67 (0.55–0.84)	0.69 (0.60–0.79)	0.485
Arg/ADMA ratio	126.0 (92.9–150.1)	110.0 (89.3–138.9)	0.211
hArg/ADMA ratio	1.62 ± 0.68	1.59 ± 0.52	0.768
Nitrate (µM)	93.2 (83.9–108.1)	89.1 (78.7–110.9)	0.444
Nitrite (µM)	3.79 (3.57–4.41)	3.96 (3.64–4.51)	0.420
P_NOx_R	23.4 (20.9–27.6)	21.6 (19.7–26.6)	0.322
MDA (µM)	0.39 (0.34–0.44)	0.39 (0.34–0.44)	0.917
Urine			
ADMA (µM/mM creatinine)	6.27 ± 4.13	6.28 ± 3.84	0.993
DMA (µM/mM creatinine)	38.7 (35.7–42.9)	37.4 (34.3–42.9)	0.262
DMA + ADMA (µM/mM creatinine)	45.5 (41.4–49.1)	44.8 (39.1–48.4)	0.502
DMA/ADMA	5.60 (4.36–13.0)	5.13 (4.46–16.2)	0.604
SDMA (µM/mM creatinine)	6.8 (5.44–8.48)	6.67 (5.24–8.55)	0.667
DMA + ADMA + SDMA (µM/mM creatinine)	53.0 (48.2–56.3)	51.7 (46.0–57.7)	0.424
(DMA + ADMA)/SDMA	6.43 (5.05–8.21)	6.67 (5.34–8.72)	0.592
Nitrate (µM/mM creatinine)	106.1 (90.0–114.1)	96.7 (78.5–128.9)	0.884
Nitrite (µM/mM creatinine)	0.35 (0.28–0.71)	0.38 (0.19–0.76)	0.786
U_NOx_R	259.2 (143.3–383.9)	287.6 (149.5–418.0)	0.631
MDA (µM/mM creatinine)	0.26 (0.20–0.45)	0.28 (0.17–0.42)	0.881
Inflammation markers			
Elevated CRP (%)	2 (5.1)	3 (6.4)	1.000 ^a^
Elevated IL-6 (%)	1 (2.4)	3 (6.4)	0.620 ^a^
Elevated IgM (%)	0 (0)	3 (6.4)	0.065 ^a^
Elevated IgG (%)	1 (2.4)	1 (2.1)	0.361 ^a^
**Elevated IgE (%)**	**29 (70.7)**	**45 (95.7)**	**0.002**

Abbreviations: AD, atopic dermatitis; ADHD, attention deficit/hyperactivity disorder; ADMA, asymmetric dimethylarginine; Arg, L-arginine; BA, bronchial asthma; DMA, dimethylamine; hArg, homoarginine; MDA, malondialdehyde; P, Plasma; P_NOx_R, nitrate/nitrite ratio in plasma; SDMA, symmetric dimethylarginine; U_NOx_R, nitrate/nitrite ratio in urine; IL-6, interleukin 6; Ig, immunoglobulin; CRP, c-reactive protein; ^a^ Fisher’s exact test. Data are presented as mean ± standard deviation (SD, normally distributed data) or median (25–75th interquartile range, non-normally distributed data). Bold *p* value indicates statistical significance.

**Table 3:** Comparison of the Arg/NO pathway in patients affected by atopic diseases or ADHD.

	Atopic Diseases	ADHD	*p* Value
Number of subjects (*n*)	95	16	-
**Plasma**			
Arg (µM)	81.7 (69.3–94.3)	75.2 (64.9–88.11)	0.271
hArg (µM)	1.13 ± 0.41	1.20 ± 0.44	0.518
ADMA (µM)	0.68 (0.58–0.82)	0.62 (0.57–0.79)	0.264
Arg/ADMA	118.4 (93.4–139.4)	115.0 (95.5–148.2)	0.675
hArg/ADMA	1.62 ± 0.63	1.96 ± 1.03	0.207
**Nitrate (µM)**	**90.1 (80.6–110.5)**	**56.6 (54.0–64.0)**	**<0.001**
**Nitrite (µM)**	**3.87 (3.62–4.56)**	**3.10 (2.95–3.26)**	**<0.001**
**P_NOx_R**	**22.6 (19.8–26.8)**	**19.0 (15.9–20.1)**	**<0.001**
MDA (µM)	0.39 (0.35–0.44)	0.39 (0.34–0.49)	0.795
**Urine**			
ADMA (µM/mM creatinine)	6.62 (2.96–9.07)	6.87 (5.97–7.60)	0.440
**DMA (µM/mM creatinine)**	**38.1 (35.0–42.93)**	**33.9 (28.9–40.6)**	**0.028**
DMA + ADMA (µM/mM creatinine)	44.8 (40.7–49.1)	41.5 (34.9–47.8)	0.084
DMA/ADMA	5.39 (4.39–13.4)	4.81 (4.17–5.38)	0.068
SDMA (µM/mM creatinine)	6.75 (5.44–8.87)	6.18 (4.29–12.8)	0.507
DMA + ADMA + SDMA (µM/mM creatinine)	52.0 (47.6–56.9)	49.1 (41.8–56.5)	0.242
(DMA + ADMA)/SDMA	6.59 (5.16–8.35)	7.16 (4.33–11.49)	0.722
Nitrate (µM/mM creatinine)	105.5 (85.3–128.2)	87.3 (75.6–114.8)	0.129
Nitrite (µM/mM creatinine)	0.39 (0.26–0.71)	0.32 (0.26–0.53)	0.369
U_NOx_R	270.1 (154.3–386.7)	259.9 (202.5–310.8)	0.940
MDA (µM/mM creatinine)	0.28 (0.19–0.43)	0.36 (0.27–0.46)	0.111
**Inflammation markers**			
Elevated CRP (%)	5 (5.4)	0 (0)	1.000 ^a^
Elevated IL-6 (%)	4 (4.2)	0 (0)	1.000 ^a^
Elevated IgM (%)	3 (3.2)	1 (6.3)	0.589 ^a^
Elevated IgG (%)	4 (4.2)	3 (18.8)	0.095 ^a^
**Elevated IgE (%)**	**81 (85.3)**	**10 (62.5)**	**0.039 ^a^**

Abbreviations: AD, atopic dermatitis; ADHD, attention deficit/hyperactivity disorder; ADMA, asymmetric dimethylarginine; Arg, L-arginine; BA, bronchial asthma; DMA, dimethylamine; hArg, homoarginine; MDA, malondialdehyde; P, Plasma; P_NOx_R, nitrate/nitrite ratio in plasma; SDMA, symmetric dimethylarginine; U, urine; U_NOx_R, nitrate/nitrite ratio in urine; IL-6, interleukin 6; Ig, immunoglobulins; CRP, C-reactive protein; ^a^ Fisher’s exact test. Data are presented as mean ± standard deviation (SD, normally distributed data) or median (25–75th interquartile range, non-normally distributed data). Bold *P* value indicates statistical significance.

**Table 4:** Comparison of Arg/NO metabolites between patients with allergy and patients without allergy.

	Patients with Allergy ^a^	Patients without Allergy	*p* Value
Number of subjects	81	30	-
**Plasma**			
Arg (µM)	79.7 (67.8–91.6)	82.2 (72.8–101.3)	0.342
hArg (µM)	1.14 ± 0.41	1.15 ± 0.43	0.935
ADMA (µM)	0.68 (0.59–0.81)	0.65 (0.58–0.83)	0.652
Arg/ADMA ratio	113.4 (91.6–139.3)	123.0 (106.9–148.0)	0.237
hArg/ADMA ratio	1.65 ± 0.66	1.72 ± 0.81	0.936
**Nitrate (µM)**	**93.2 (80.1–110.7)**	**75.0 (57.1–87.3)**	**<0.001**
**Nitrite (µM)**	**4.07 ± 0.66**	**3.58 ± 0.57**	**<0.001**
P_NOx_R	22.2 (19.6–26.7)	20.8 (18.2–23.8)	0.098
MDA (µM)	0.38 (0.35–0.44)	0.39 (0.33–0.43)	0.498
Urine			
ADMA (µM/mM creatinine)	6.17 ± 3.88	7.24 ± 3.25	0.183
DMA (µM/mM creatinine)	38.0 (35.1–43.2)	37.2 (31.3–41.8)	0.123
DMA + ADMA (µM/mM creatinine)	45.0 (41.0–48.9)	43.4 (38.6–48.2)	0.321
DMA/ADMA ratio	5.44 (4.47–13.5)	4.81 (3.97–6.40)	0.053
SDMA (µM/mM creatinine)	6.71 (5.28–8.82)	6.59 (4.79–10.8)	0.765
DMA + ADMA + SDMA(µM/mM creatinine)	52.6 (47.6–57.8)	50.4 (45.5–54.1)	0.193
(DMA + ADMA)/SDMA ratio	6.67 (5.22–8.66)	6.69 (4.78–9.20)	0.825
Nitrate (µM/mM creatinine)	101.3 (83.3–128.4)	108.2 (78.8–116.1)	0.666
Nitrite (µM/mM creatinine)	0.36 (0.26–0.71)	0.33 (0.27–0.67)	0.942
U_NOx_R	287.6 (163.0–401.2)	249.9 (160.4–312.8)	0.377
MDA (µM/mM creatinine)	0.29 (0.18–0.45)	0.30 (0.23–0.45)	0.459
**Inflammation markers**			
Elevated CRP (%)	5 (6.2)	0 (0)	0.325 ^b^
Elevated IL-6 (%)	4 (4.9)	0 (0)	0.573 ^b^
Elevated IgM (%)	3 (3.7)	1 (3.3)	0.458 ^b^
Elevated IgG (%)	4 (4.9)	3 (10.0)	0.323 ^b^
**Elevated IgE (%)**	**78 (96.3)**	**13 (43.3)**	**<0.001**

Abbreviations: AD, atopic dermatitis; ADHD, attention deficit/hyperactivity disorder; ADMA, asymmetric dimethylarginine; Arg, L-arginine; BA, bronchial asthma; DMA, dimethylamine; hArg, homoarginine; MDA, malondialdehyde; P_NOx_R, nitrate/nitrite ratio in plasma; SDMA, symmetric dimethylarginine; U_NOx_R, nitrate/nitrite ratio in urine; IL-6, interleukin 6; Ig, immunoglobulin; CRP, C-reactive protein; ^a^ Allergy was defined as specific IgE over 3.5 U/mL; ^b^ Fisher’s exact test. Data are presented as mean ± standard deviation (SD, normally distributed data) or median (25–75th interquartile range, non-normally distributed data). Bold *P* value indicates statistical significance.

**Table 5:** Comparison of the Arg/NO pathway between atopic patients with allergy and atopic patients without allergy.

	BA, AD, and BA + AD Patients		*p* Value
	With Allergy ^a^	Without Allergy	
Number of subjects	78	17	-
**Plasma**			
Arg (µM)	79.7 (68.0–92.0)	86.4 (77.8–108.3)	0.072
hArg (µM)	1.14 ± 0.41	1.09 ± 0.40	0.650
ADMA (µM)	0.69 (0.59–0.82)	0.66 (0.56–0.80)	0.472
**Arg/ADMA ratio**	**112.5 (91.0–137.9)**	**135.3 (115.0–152.1)**	**0.037**
hArg/ADMA ratio	1.62 ± 0.65	1.59 ± 0.51	0.830
Nitrate (µM)	93.7 (80.5–110.6)	85.8 (82.0–108.0)	0.669
**Nitrite (µM)**	**4.00 (3.63–4.59)**	**3.72 (3.58–3.85)**	**0.040**
P_NOx_R	22.4 (19.7–26.6)	22.7 (21.0–28.9)	0.443
MDA (µM)	0.39 (0.35–0.44)	0.39 (0.33–0.41)	0.273
**Urine**			
ADMA (µM/mM creatinine)	6.13 ± 3.95	7.05 ± 3.80	0.382
DMA (µM/mM creatinine)	38.1 (35.1–43.3)	38.2 (33.3–42.0)	0.452
DMA + ADMA (µM/mM creatinine)	45.0 (40.6–49.1)	44.1 (40.3–48.6)	0.764
DMA/ADMA ratio	5.58 (4.48–14.9)	4.73 (3.62–7.81)	0.180
SDMA (µM/mM creatinine)	6.66 (5.22–8.60)	7.17 (6.15–11.1)	0.240
DMA + ADMA + SDMA (µM/mM creatinine)	52.2 (47.4–57.7)	50.6 (48.6–54.4)	0.764
(DMA + ADMA)/SDMA ratio	6.73 (5.36–8.73)	5.53 (4.41–7.65)	0.113
Nitrate (µM/mM creatinine)	102.0 (83.9–128.3)	109.4 (89.1–129.4)	0.466
Nitrite (µM/mM creatinine)	0.39 (0.25–0.70)	0.60 (0.29–0.79)	0.332
U_NOx_R	289.5 (159.12–400.01)	208.0 (144.7–331.5)	0.332
MDA (µM/mM creatinine)	0.26 (0.17–0.45)	0.29 (0.2–0.43)	0.844
**Inflammation markers**			
Elevated CRP (%)	5 (6.4)	0 (0)	0.588 ^b^
Elevated IL-6 (%)	4 (5.1)	0 (0)	1.000 ^b^
Elevated IgM (%)	3 (3.8)	0 (0)	0.159 ^b^
Elevated IgG (%)	3 (3.8)	1 (5.9)	0.250 ^b^
**Elevated IgE (%)**	**75 (96.2)**	**6 (35.3)**	**<0.001 ^b^**

Abbreviations: AD, atopic dermatitis; ADHD, attention deficit/hyperactivity disorder; ADMA, asymmetric dimethylarginine; Arg, L-arginine; BA, bronchial asthma; DMA, dimethylamine; hArg, homoarginine; MDA, malondialdehyde; P, Plasma; P_NOx_R, nitrate/nitrite ratio in plasma; SDMA, symmetric dimethylarginine; U, urine; U_NOx_R, nitrate/nitrite ratio in urine; IL-6, interleukin 6; Ig, immunoglobulin; CRP, C-reactive protein; ^a^ Allergy was defined as specific IgE over 3.5 U/mL; ^b^ Fisher’s exact test. Data are presented as mean ± standard deviation (SD, normally distributed data) or median (25–75th interquartile range, non-normally distributed data). Bold *p* value indicates statistical significance.

## Supplementary Materials

The following supporting information can be downloaded at: https://www.mdpi.com/article/10.3390/ijms23042136/s1.
