# Supplementary material for: Characterization of the L-Arginine/Nitric Oxide Pathway and Oxidative Stress in Pediatric Patients with Atopic Diseases
Source: Int J Mol Sci. 2022 Feb 15;23(4):2136. doi: 10.3390/ijms23042136 (PMC8878534; doi:10.3390/ijms23042136)
Supplement: Supplementary file 1 [file ijms-23-02136-s001.zip › ijms-1586047-supplementary.pdf]

International Journal of *Molecular Sciences*

MDPI

Article

Characterization of the L-Arginine/Nitric Oxide Pathway and Oxidative Stress in Pediatric Patients with Atopic Diseases

Beatrice Hanusch [1], Kathrin Sinningen [1], Folke Brinkmann [1], Stefanie Dillenhöfer [1], Mirjam Frank [2], Karl-Heinz Jöckel [2], Cordula Koerner-Rettberg [3], Martin Holtmann [4], Tanja Legenbauer [4], Christian Langrock [5], Thomas Reinehr [5], Patricia Maasjosthusmann [6], Bibiana Beckmann [7], Eckard Hamelmann [6,†], Dimitrios Tsikas [7,†] and Thomas Lücke [1,*,†]

[1] University Hospital of Pediatrics and Adolescent Medicine, St. Josef-Hospital, Ruhr-University Bochum, 44791 Bochum, Germany; beatrice.hanusch@rub.de (B.H.); kathrin.sinningen@rub.de (K.S.); folke.brinkmann@rub.de (F.B.); stefanie.dillenhoefer@klinikum-bochum.de (S.D.)
[2] Institute for Medical Informatics, Biometry and Epidemiology, University of Duisburg-Essen, 45122 Essen, Germany; mirjam.frank@uk-essen.de (M.F.); k-h.joeckel@uk-essen.de (K.-H.J.)
[3] Clinic for Children and Youth Medicine, Marien-Hospital, 46483 Wesel, Germany; cordula.koerner-rettberg@prohomine.de
[4] LWL University Hospital for Child and Adolescent Psychiatry, Ruhr University Bochum, 44791 Bochum, Germany; martin.holtmann@lwl.org (M.H.); tanja.legenbauer@rub.de (T.L.)
[5] Department of Pediatric Endocrinology, Diabetes and Nutrition Medicine, Vestische Kinder- und Jugendklinik Datteln, University of Witten/Herdecke, 45711 Datteln, Germany; info@psychotherapie-langrock.de (C.L.); t.reinehr@kinderklinik-datteln.de (T.R.)
[6] University Children's Center Bethel, Protestant Hospital Bethel, University Bielefeld, 33617 Bielefeld, Germany; patricia.maasjosthusmann@evkb.de (P.M.); eckard.hamelmann@evkb.de (E.H.)
[7] Institute of Toxicology, Core Unit Proteomics, Hannover Medical School, 30623 Hannover, Germany; beckmann.bibiana@mh-hannover.de (B.B.); tsikas.dimitros@mh-hannover.de (D.T.)
* Correspondence: thomas.luecke@ruhr-uni-bochum.de; Tel.: +49-234-509-2611
† Shared senior authors.

Keywords: Atopic diseases; nitric oxide; attention deficit/hyperactivity syndrome; bronchial asthma; atopic dermatitis; common pediatric diseases

Citation: Hanusch, B.; Sinningen, K.; Brinkmann, F.; Dillenhöfer, S.; Frank, M.; Jöckel, K.-H.; Koerner-Rettberg, C.; Holtmann, M.; Legenbauer, T.; Langrock, C.; et al. Characterization of the L-Arginine/Nitric Oxide Pathway and Oxidative Stress in Pediatric Patients with Atopic Diseases. *Int. J. Mol. Sci.* **2022**, *23*, 2136. https://doi.org/10.3390/ijms23042136

Academic Editors: Madhav Bhatia and Isao Ishii

Received: 21 January 2022
Accepted: 9 February 2022
Published: 15 February 2022

Publisher's Note: MDPI stays neutral with regard to jurisdictional claims in published maps and institutional affiliations.

Copyright: © 2022 by the authors. Licensee MDPI, Basel, Switzerland. This article is an open access article distributed under the terms and conditions of the Creative Commons Attribution (CC BY) license (https://creativecommons.org/licenses/by/4.0/).

Table S1 Comparison between pediatric patients only affected by BA, AD or by a combination of BA and AD

	BA	AD	BA + AD	*P* Value
Number of subjects (n)	41	7	47	-
**Plasma**				
Arg (P) (μM)	84.07 (71.15 – 99.10)	77.75 (73.73 – 113.71)	81.74 (66.96 – 91.46)	0.586
hArg P (μM)	1.14 ± 0.49	1.28 ± 0.54	1.11 ± 0.29	0.583
ADMA (P) (μM)	0.67 (0.55 – 0.84)	0.77 (0.55 – 0.88)	0.69 (0.60 – 0.79)	0.626
Arg/ADMA ratio (P)	125.97 (92.89 – 150.11)	111.58 (104.23 – 136.27)	109.98 (89.25 – 138.93)	0.415
hArg/ADMA	1.62 ± 0.68	1.79 ± 1.00	1.59 ± 0.52	0.728
Nitrate (P) (μM)	93.24 (83.91 – 108.05)	84.44 (76.17 – 112.98)	89.07 (78.70 – 110.85)	0.641
Nitrite (P) (μM)	3.79 (3.57 – 4.41)	4.56 (3.70 – 5.19)	3.96 (3.64 – 4.51)	0.276
$P_{NOx}R$	23.41 (20.85 – 27.60)	19.41 (16.72 – 23.55)	21.58 (19.73 – 26.55)	0.216
**Urine**				
ADMA (μM/mM creatinine)	6.27 ± 4.13	6.51 ± 3.76	6.28 ± 3.84	0.989
DMA (μM/mM creatinine)	38.68 (35.66 – 42.91)	41.97 (34.63 – 55.69)	37.35 (34.33 – 42.93)	0.317
DMA + ADMA	45.52 (41.41 – 49.09)	44.27 (42.94 – 66.20)	44.81 (39.12 – 48.35)	0.664
DMA/ADMA	5.60 (4.36 – 12.96)	6.48 (4.40 – 13.62)	5.13 (4.46 – 16.19)	0.794
SDMA (μM/mM creatinine)	6.8 (5.44 – 8.48)	9.00 (5.51 – 10.72)	6.67 (5.24 – 8.55)	0.660
DMA + ADMA + SDMA	53.04 (48.23 – 56.31)	54.39 (48.45 – 75.20)	51.67 (45.97 – 57.68)	0.559
(DMA + ADMA)/SDMA	6.43 (5.05 – 8.21)	6.96 (4.26 – 7.79)	6.67 (5.34 – 8.72)	0.845
Nitrate (μM/mM creatinine)	106.06 (90.01 – 114.07)	136.41 (88.36 – 198.84)	96.66 (78.52 – 128.86)	0.132
Nitrite (μM/mM creatinine)	0.35 (0.28 – 0.71)	0.59 (0.24 – 1.03)	0.38 (0.19 – 0.76)	0.809
$U_{NOx}R$	259.20 (143.32 – 383.91)	216.36 (162.39 – 350.80)	287.57 (149.48 – 418.02)	0.888

Abbreviations: AD, atopic dermatitis; ADHD, Attention Deficit/Hyperactivity Disorder; ADMA, asymmetric dimethylarginine; Arg, L-arginine; BA, bronchial asthma; DMA, dimethylamine; hArg, homoarginine; P, Plasma; $P_{NOx}R$, nitrate/nitrite ratio in plasma; SDMA, symmetric dimethylarginine; U, urine; $U_{NOx}R$, nitrate/nitrite ratio in urine. Data are presented as mean ± standard deviation (SD, normally distributed data) or median (25-75th interquartile range, non-normally distributed data).

Table S2 Comparison of NO metabolite concentration between pediatric patients with well-controlled asthma bronchiale (BA) and such with not-well controlled BA, classified according to the asthma control test [71,72]

	BA well controlled	BA not-well controlled	*P* Value
Number of subjects (*n*)	72	20	-
Asthma control test scores	24 (22 – 26)	17 (15 – 18)	-
	**Plasma**		
Arg (µM)	81.11 (67.81 – 92.71)	84.94 (69.54 – 99.32)	0.589
hArg (µM)	1.14 ± 0.43	1.12 ± 0.33	0.758
ADMA (µM)	0.68 (0.59 – 0.82)	0.65 (0.55 – 0.78)	0.460
Arg/ADMA ratio	118.76 (93.59 – 141.17)	120.31 (104.03 – 145.88)	0.389
hArg/ADMA ratio	1.63 ± 0.66	1.69 ± 0.55	0.729
Nitrate (µM)	87.50 (79.05 – 108.26)	92.42 (84.37 – 112.04)	0.248
Nitrite (µM)	3.81 (3.57 – 4.44)	4.13 (3.85 – 4.58)	0.074
$P_{NOx}R$	22.65 (19.75 – 26.57)	21.35 (19.85 – 27.48)	0.748
MDA (µM)	0.39 (0.35 - 0.43)	0.47 (0.36 - 0.67)	0.478
	**Urine**		
ADMA (µM/mM creatinine)	6.53 ± 3.94	5.67 ± 3.90	0.389
DMA (µM/mM creatinine)	38.22 (35.11 – 42.85)	36.74 (34.32 – 43.02)	0.466
DMA + ADMA	45.30 (41.13 – 49.02)	43.44 (39.92 – 49.05)	0.478
DMA/ADMA	5.30 (4.40 – 10.68)	5.68 (4.35 – 17.62)	0.694
SDMA	6.68 (5.47 – 8.84)	6.91 (5.18 – 8.91)	0.962
DMA + ADMA + SDMA	51.92 (47.70 – 56.61)	53.34 (46.53 – 57.71)	0.908
(DMA + ADMA)/SDMA	6.52 (5.17 – 7.87)	6.59 (4.83 – 8.76)	0.916
Nitrate (µM/mM creatinine)	106.31 (84.71 – 127.87)	95.37 (86.45 – 129.45)	0.762
Nitrite (µM/mM creatinine)	0.38 (0.26 – 0.68)	0.50 (0.26 – 1.00)	0.421
$U_{NOx}R$	281.99 (163.43 – 374.09)	247.07 (110.50 – 410.19)	0.570
MDA (µM /mM creatinine)	0.28 (0.20 - 0.46)	0.25 (0.18 - 0.55)	0.887
	**Inflammation**		
IGA	136.23 ± 53.25	153.30 ± 47.81	0.199
IGE	395.00 (105.00 – 863.00)	793.00 (242.00 – 1137.25)	0.084
IGG	1018.73 ± 193.25	1095.45 ± 194.01	0.121
IGM	84.00 (65.00 – 115.00)	92.50 (52.50 – 122.00)	0.893
IL6	1.80 (1.20 – 2.45)	1.60 (1.40 – 2.40)	0.634

Abbreviations: AD, atopic dermatitis; ADMA, asymmetric dimethylarginine; Arg, L-arginine; BA, bronchial asthma; DMA, dimethylamine; hArg, homoarginine; P, Plasma; $P_{NOx}R$, nitrate/nitrite ratio in plasma; SDMA, symmetric dimethylarginine; U, urine; $U_{NOx}R$, nitrate/nitrite ratio in urine. Data are presented as mean ± standard deviation (SD, normally distributed data) or median (25-75th interquartile range, non-normally distributed data).

Table S3 Comparison of concentrations of NO metabolites in patients affected by light to moderate atopic dermatitis (AD) and severe AD classified according to SCORAD

	Moderate AD	Severe AD	*P* Value
Number of subjects (*n*)	36	9	-
**Plasma**			
Arg (µM)	83.65 ± 22.34	87.5 ± 16.06	0.631
hArg (µM)	1.15 ± 0.34	1.1 ± 0.39	0.721
ADMA (µM)	0.69 (0.6 - 0.81)	0.68 (0.58 - 0.74)	0.770*
Arg/ADMA ratio	117.27 ± 34.68	131.79 ± 27.69	0.251
hArg/ADMA ratio	1.62 ± 0.55	1.71 ± 0.78	0.681
Nitrate (µM)	93.01 ± 19.79	90.63 ± 16.59	0.742
Nitrite (µM)	4.07 ± 0.58	4.19 ± 1.06	0.621
$P_{NOx}R$	21.03 (19.03 - 26.48)	21.76 (19.13 - 26.77)	0.856*
MDA (µM)	0.38 (0.36 - 0.43)	0.37 (0.33 - 0.59)	0.989
**Urine**			
ADMA (µM/mM creatinine)	6.58 (3 - 8.66)	6.79 (3.47 - 8.67)	0.727*
DMA (µM/mM creatinine)	37.36 (34.5 - 42.85)	37.85 (31.82 - 41.86)	0.753*
DMA + ADMA	44.27 (40.38 - 47.66)	42.03 (37.38 - 48.54)	0.710*
DMA/ADMA	5.3 (4.73 - 13.62)	4.52 (4.38 - 12.56)	0.439*
SDMA	6.88 (5.3 - 8.98)	5.32 (2.96 - 9.37)	0.211*
DMA + ADMA + SDMA	50.62 (46.54 - 56.19)	47.7 (40.51 - 57.91)	0.358*
(DMA + ADMA)/SDMA	6.72 (5.17 - 7.66)	8.77 (5.39 - 14.08)	0.165*
Nitrate (µM/mM creatinine)	113.67 (78.38 - 134.5)	94.55 (71.22 - 190.46)	> 0.999*
Nitrite (µM/mM creatinine)	0.38 (0.19 - 0.65)	0.37 (0.26 - 1.05)	0.791*
$U_{NOx}R$	302.47 (159.3 - 421.9)	265.94 (199.38 - 351.87)	0.856*
MDA (µM /mM creatinine)	0.28 (0.19 - 0.46)	0.24 (0.12 - 0.35)	0.181
**Inflammation**			
IGA	154.19 ± 51.96	128.22 ± 26.36	0.156
IGE	304.00 (135.75 - 900.25)	581 (341 - 2534.5)	0.131*
IGG	1081.08 ± 186.82	1062.56 ± 153.29	0.785
IGM	104.47 ± 49.8	88.44 ± 27.35	0.360
IL6	2.00 (1.18 - 2.58)	1.9 (1.45 - 3.05)	0.758*

Abbreviations: AD, atopic dermatitis; ADMA, asymmetric dimethylarginine; Arg, L-arginine; BA, bronchial asthma; DMA, dimethylamine; hArg, homoarginine; P, Plasma; $P_{NOx}R$, nitrate/nitrite ratio in plasma; SDMA, symmetric dimethylarginine; U, urine; $U_{NOx}R$, nitrate/nitrite ratio in urine, *exact significance. Data are presented as mean ± standard deviation (SD, normally distributed data) or median (25-75th interquartile range, non-normally distributed data).

Table S4 Spearman correlation of immunoglobulin E (IgE) concentration in plasma and nitrate and nitrite in plasma and urine

**IgE vs.**	**Correlation coefficient r**	***P* value**
	Plasma	
Nitrate (μM)	0.065	0.501
Nitrite (μM)	0.147	0.139
	Urine	
Nitrate (μM/mM creatinine)	0.008	0.938
Nitrite (μM/mM creatinine)	<0.001	0.998
